# Investigation of X-rays Beams Uniformity in Radiotherapeutic Tumor Treatment Procedure Using LuAG:Ce Crystal Detectors

**DOI:** 10.3390/ma17164016

**Published:** 2024-08-13

**Authors:** Sandra Witkiewicz-Łukaszek, Janusz Winiecki, Paulina Michalska, Seweryn Jakubowski, Oleg Sidletskiy, Yuriy Zorenko

**Affiliations:** 1 Faculty of Physics, Kazimierz Wielki University in Bydgoszcz, Powstańców Wielkopolskich Street 2, 85-090 Bydgoszcz, Poland; 2 Franciszek Łukaszyk Oncology Center, Medical Physics Department, dr Izabeli Romanowskiej Street 2, 85-796 Bydgoszcz, Poland; p.michalska@o2.pl (P.M.); jakubowskis@co.bydgoszcz.pl (S.J.); 3 Department of Oncology and Brachytherapy, Collegium Medicum in Bydgoszcz of Nicholas Copernicus University in Toruń, Jagiellońska Street 13/15, 85-067 Bydgoszcz, Poland; 4 Institute of Scintillation Materials, National Academy of Sciences of Ukraine, Av. Nauki 60, 61178 Kharkiv, Ukraine; sidletskiy@isma.kharkov.ua

**Keywords:** thermoluminescence, LuAG:Ce crystals, X- and γ-rays, radiotherapy

## Abstract

In this study, Ce^3+^-doped Lu_3_Al_5_O_12_ garnet (LuAG) crystal detectors, with a density of ρ = 6 g/cm^3^ and an effective atomic number Z_eff_ = 62, are proposed as promising materials for radiotherapy applications. This type of detector demonstrates excellent uniformity of structural and optical properties, high thermoluminescence (TL) light yield, optimal position of main TL glow peaks at temperatures around 245–295 °C, and high radiation stability. The set of TL detectors made from LuAG:Ce single crystal was used to evaluate the uniformity of dose and energy spectra of X-ray radiation from a clinical accelerator with 6 MV and 15 MV beams at the Department of Medical Physics, Oncology Center in Bydgoszcz, Poland, and γ-rays from a ^60^Co source at the National Institute of Oncology in Warsaw. The LuAG:Ce crystal detectors demonstrated highly promising results for registering X-ray radiation from accelerators with both 6 MV and 15 MV electron beams, as well as γ-rays from a ^60^Co source with energies of 1.17 and 1.33 MeV.

## 1. Introduction

The primary objective of radiotherapy is to administer ionizing radiation to cancerous tissue, inducing the destruction of cancer cells. However, for effective treatment planning and customized dose distribution, a precise understanding of therapeutic beam quality is essential. Two key dosimetric parameters, uniformity and flatness, determine the characteristics of radiation intensity (dose) in the plane perpendicular to the beam axis [1,2,3,4,5,6,7]. An ideal dosimeter for radiotherapy should possess several key features: a low detection limit, high accuracy and precision, the ability to detect radiation over a wide dose range, a linear dose-response that is independent of dose rate and radiation energy, and the capability to measure doses in very small volumes, ensuring high spatial resolution.

Nowadays, the conventional method of measuring the dose of an X-ray beam in radiotherapy involves the use of large-sized ionization cameras and semiconductor detectors. However, such a dosimetric approach is very difficult to implement in the case of precise estimation of the dose and energy distribution of high-energy X- and γ-ray beams above 1 MeV. This problem determines the use of other dosimetric methods and materials.

Thermostimulated luminescence (TL) dosimetry serves as a versatile method for evaluating doses of different types of ionizing radiation. Various TL detectors, primarily composed of LiF and Al_2_O_3_:C compounds, are typically used for this purpose. However, due to the very high energies of X-ray beams produced by linear electron accelerators (linac), usually working with 6 MV and 15 MV of nominal accelerating potential (NAP), application of the conventional TL detectors based on LiF or Al_2_O_3_:C is limited. That is also true in the case of the application of radiation treatment with the conventional ^60^Co γ-rays source with typical energies of 1.17 and 1.33 MeV. Assuming that the X-and γ-ray absorption ability of the materials is proportional to ρ × Z_eff_^4^, such limitations correspond mainly to the low-density ρ and effective atomic number Z_eff_ of the mentioned TL materials.

Taking into account the above-mentioned challenges, novel TL materials based on crystals, crystal-film composites, and ceramics derived from established oxide materials, showcasing a broad spectrum of ρ × Z_eff_^4^ values, emerge as more promising candidates for TL detectors in radiotherapy contexts [8,9,10,11,12,13,14,15]. These innovative materials, crucial for ensuring dose radiation uniformity in routine diagnostic and therapeutic procedures, must effectively absorb ionizing radiation across a wide energy spectrum. This includes conventional X-ray sources with energy ranging from 5 to 80 keV, as well as high-energy 6 MV and 15 MV beams from typical linac accelerators, as well as gamma rays with energies of 392 keV or 1.17 and 1.33 MeV generated by therapeutic γ-ray sources such as ^192^Ir or ^60^Co, respectively.

As possible candidates for new TL materials, we considered in our recent studies [9,10,11,12,13,14,15] the well-known rare-earth doped A_3_B_5_O_12_ (A = Y, Lu, Gd; B = Al, Ga) garnets are widely used as laser media, scintillators, and cathodoluminescence screens. Due to the well-developed production technologies of these garnets in the crystal, film, and composite forms, very high material uniformity can be obtained in the case of preparation of large sets of TL detectors for diagnostic of the uniformity of therapeutic X-ray and γ-ray sources.

Single crystals of Ce^3+^-doped Y_3_Al_5_O_12_ (YAG), Lu_3_Al_5_O_12_ (LuAG:Ce), and Gd_3_Al_2_Ga_3_O_12_ (GAGG) garnets are currently considered for applications as fast and efficient scintillators due to their excellent radiation stability, high yield (20–25 Ph/KeV), and short decay time (50–70 ns) [16,17,18,19]. However, the YAG:Ce and LuAG:Ce crystals are also characterized by large content (even up to 0.1–25 at.%) of Y_Al_ or Lu_Al_ antisite defects (ADs) and oxygen vacancies as a consequence of high-temperature (1970–2030 °C) growth of bulk crystals of these garnets from melt in the inert (Ar) or vacuum atmosphere [20,21,22,23,24]. The Y_Al_ and Lu_Al_ ADs and charged oxygen vacancies in the YAG and LuAG crystals act as effective emission centers in the UV range and trapping centers as well [25,26,27,28,29,30,31].

For this reason, apart from the scintillation applications, the crystals of YAG:Ce garnet with ρ = 4.5 g/cm^3^; Z_eff_ = 35 and crystals of LuAG:Ce garnet with ρ = 6.75 g/cm^3^; Z_eff_ = 59, considered recently as possible alternative materials for TL dosimetry due to high TL signal and good position of main glow peak in the 200–300 K range after irradiation with various ionization radiation sources [8,9,12,14,27]. 

Furthermore, the thermoluminescence (TL) and optically stimulated luminescence (OSL) characteristics of YAG:Ce, LuAG:Ce, and GAGG:Ce crystals have been extensively examined in works [9,10,11,12,13,14,15]. These crystals exhibit several unicity properties that put them very high on the list of promising candidates for radiation- and chemical-resistant detectors with small volume and high spatial resolution [10,12,14]. Moreover, using the liquid phase epitaxy (LPE) growth technique, advanced *composite TL detectors* have been developed. These detectors feature film-crystal epitaxial structures of YAG:Ce and LuAG:Ce garnets, enabling simultaneous detection of various components of mixed ionizing radiation [13,14]. The separate detection of different radiation types in these composite detectors occurs due to differences between the TL glow curves after α- and β-particles or γ-ray excitations, which are recorded from both the film and substrate parts of YAG:Ce film/LuAG:Ce crystal and LuAG:Ce film/YAG:Ce crystal composite detectors [13].

In this study, we used LuAG garnet crystals doped by Ce^3+^ with specific TL properties for clinical dosimetry applications. Our aim was to assess the uniformity of dose and energy spectra of X-ray radiation generated by accelerators with 6 MV and 15 MV beams at the Radiotherapy Department of the Oncology Center in Bydgoszcz, Poland, as well as from a ^60^Co γ-ray source with energies of 1.17 and 1.33 MeV at the National Institute of Oncology in Warsaw, Poland.

## 2. Samples and Equipment

To test the uniformity of an X-ray beam, we used a set of LuAG:Ce TL detectors prepared from the same Czochralski-grown crystal. Four samples of LuAG:Ce crystals, each with 5 mm × 5 mm × 1 mm dimension and exhibiting similar cathodoluminescence (CL) and TL properties, were selected. These detectors were irradiated with low-energy X-rays (40–140 kV), high-energy X-rays (6 MV and 15 MV), and high-energy γ-rays (1.17 and 1.33 MeV) from a ^60^Co source. For low-energy X-rays, an Acuity radiotherapy simulator was used, while for high-energy X-rays, a 2300C/D Clinac linear accelerator was employed. Both devices, from Varian Medical Systems (Palo Alto, CA, USA), are operated at the Radiotherapy Department of the Oncology Center in Bydgoszcz. Exposure to γ-ray radiation was conducted at the Department of Medical Physics, National Institute of Oncology in Warsaw, using a Theratron 780C setup from Best Theratronics Ltd. (Kanata, ON, Canada).

**Figure 1:**
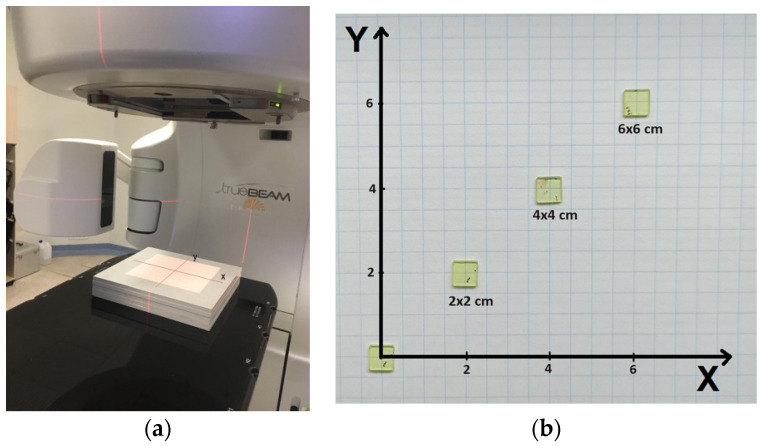
Setup for LuAG:Ce detectors irradiation: (**a**) RW3 solid water slab phantom (PTW dr Pychlau GmbH, Freiburg im Breisgau, Germany) set in the isocenter of TrueBeam linear accelerator (Varian Medical System, Palo Alto, CA, USA), (**b**) photo of LuAG:Ce detectors and their positions (in cm) identified by coordinates in the x-y plane.

The location of the set of LuAG:Ce detectors on the therapeutic table is shown in Figure 1a. The samples are positioned at 0 × 0 cm, 2 × 2 cm, 4 × 4 cm, and 6 × 6 cm from the center of the axis (Figure 1b).

The CL spectra of LuAG:Ce crystals in the 200–800 nm range were recorded at room temperature (RT) using spectrometer Steller Net Silver Nova TE-cooled CCD detector with grating monochromator (StellerNet Inc., Tampa, FL, USA) under e-beam excitation from electron microscope SEM JEOL KSM–6400 (Akishima, Japan).

The TL glow curves of LuAG:Ce detectors after exposure to various types of ionizing radiation were measured using a TL-reader (produced by the Institute of Physics of the Polish Academy of Science, Warsaw, Poland). The TL glow curves were recorded with a heating rate of 1 degree per second, with a final heating temperature set at 400 °C. Consistent procedures were implemented for each measurement cycle, ensuring uniformity in the duration between irradiation and measurement. The detectors were consistently stored under controlled conditions to mitigate the impact of external factors such as sunlight, temperature, and humidity on signal stability. Experimental errors in determining the TSL glow peak position and intensity were estimated at approximately 1.5–2 °C and 3–5%, respectively.

Finally, the spectra of the thermostimulated luminescence (TSL) of X-ray irradiated LuAG:Ce crystals with a dose of 2 Gy were recorded using a very sensitive spectrometer Avantes HERO (Avantes B.V. Apeldoorn, The Netherlands) at heating in the vicinity of main TL peaks at the 240–300 °C range. A Schott BG 39 green filter (Schott AG, Wolverhampton, UK) was used in all measurements to separate the Ce^3+^ luminescence from LuAG:Ce detectors. This filter’s transmittance range, extending from 450 to 700 nm, matched well with the emission range of Ce^3+^ emission in the LuAG:Ce crystals. Given that Ce^3+^ ions typically act as hole-trapping centers in TL processes in oxides, all the observed peaks in the TL glow curves of the LuAG:Ce detectors are attributed to electron-trapping centers. Meanwhile, in garnet crystals grown from high-temperature melts, such centers are typically created by Lu_Al_ antisite defects (ADs), oxygen vacancies, and their aggregates as well (see [25,32] for details). To register the luminescence of these defect centers in TSL processes, the TL glow curves and TSL spectra were also recorded using a UV 340 filter, which has a transparency range of 250–420 nm.

## 3. Experimental Setups for Irradiation and Sources of Ionizing Radiation

Currently, high-energy X-ray radiation generated by linear accelerators is most often used in oncological External Beam Radiotherapy (EBRth) treatment. Less often, gamma radiation (usually used in brachytherapy) and charged particles (protons, electrons, and heavy ions) are used. Gamma radiation is used primarily in brachytherapy (^192^Ir isotope), although in EBRth, the classic ^60^Co sources are still used in stereotactic irradiation of brain tumors (Gamma Knife). Therapy with charged particles (protons, electrons, heavy ions) and low-energy X-rays are also carried out, but to a lesser extent.

For this reason, three types of ionizing radiation were used in the study: (i) continuous low-energy X-ray sources operating at accelerating voltages ranging from 40 to 140 kV; (ii) γ-rays with energies of 1.17 and 1.33 MeV from a ^60^Co isotope; and (iii) high-energy X-rays generated by a clinical accelerator using electron beams accelerated at 6 MV and 15 MV potentials [33].

**Figure 2:**
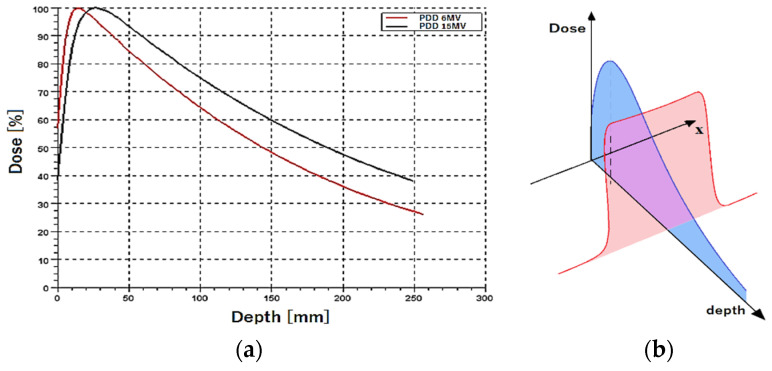
Characteristics of the high energy X-ray beam in EBRth: (**a**) transversal profile for 6 and 15 MV beams, (**b**) percentage depth-dose distribution (blue curve) and typical beam profile for radiotherapeutic applications (red curve).

The therapeutic effect is achieved by delivering to the indicated tissue area (understood as a habitat of cancer cells) a prescribed uniform dose of radiation. In EBRth, this is possible, among other things, by monitoring the quality and uniformity of the accelerator radiation beam. Both the flatness expressing the transverse uniformity of the (transversal profile), and the percentage depth-dose distribution (PDD) are controlled (Figure 2).

**Figure 3:**
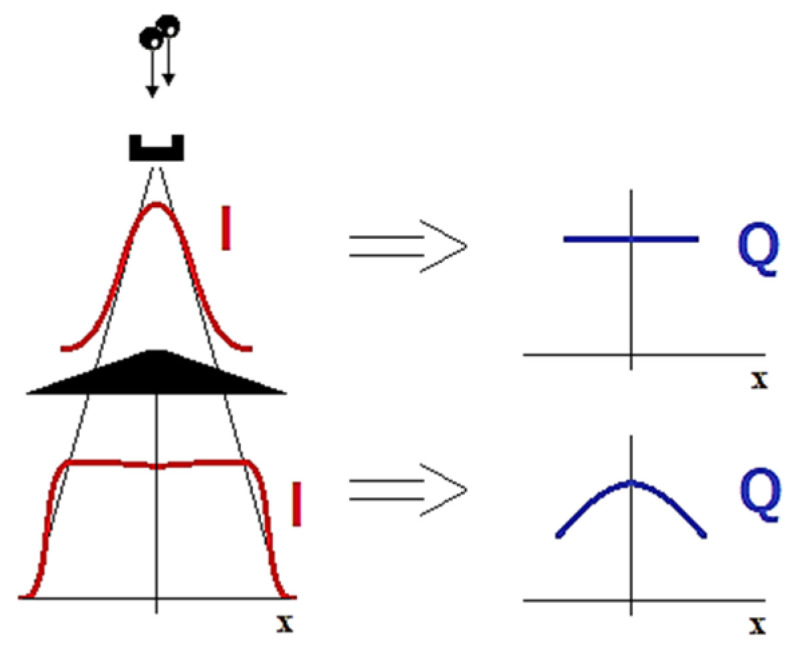
Flattening of the high-energy X-ray beam in conventional accelerator and expected consequences in beam spectrum. Symbol I means beam intensity, and Q is the quality of radiation.

For many years, beam uniformity was achieved simply by using a *flattening filter* permanently installed in the accelerator head. In the central part of the beam profile, the intensity was significantly reduced. This caused the original beam to obtain a stable, approximately flat profile, but it also led to some changes in the beam’s energetic spectrum. The peripheral areas of the therapeutic beam had a virtually unchanged spectrum, while in the central axis, the radiation was much harder due to the use of a filter (Figure 3). The flattening filter is necessary because, without a flattening filter, the X-ray or electron beam produced by the accelerator would have a non-uniform intensity, e.g., being more intense at the center and less intense at the edges. This natural Gaussian distribution is not suitable for treating tumors effectively, as it could lead to uneven dose delivery. 

**Figure 4:**
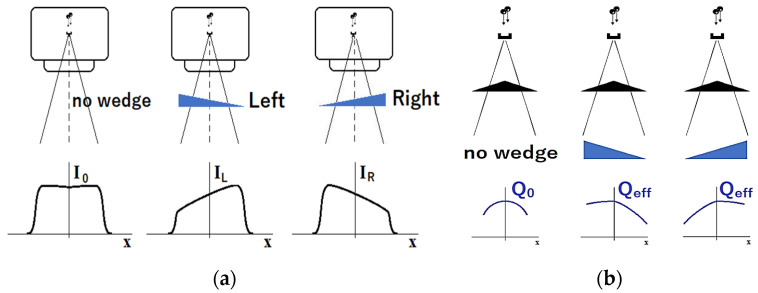
(**a**) Explanation of influence of wedge filters on dose distribution: no filter (**left**), Left_60° wedge (**middle**) and Rigth_60° wedge (**right**) filters for C-arm linear accelerator from Varian Medical Systems. I_0_—intensity for open beam, I_L_ and I_R_—intensity for left and right orientation of wedge. (**b**) effect of flattening filter and wedge filters on transversal intensity and effective quality of radiation: Q_0_—original quality distribution, Q_eff_—effective quality distribution.

In routine clinical work, additional left- and right-oriented wedge filters are sometimes used. The wedge filters allow for additional modification of the dose distribution in the patient if necessary (Figure 4a). The filters are made of steel, copper, iron, or other metals with high density and high atomic number. They also have their own impact on the modification of the radiation spectrum. In this work, we used different oriented wedge filters to reduce (right filter) and/or enhance (left filter) the effect of the flattening filter on modifying the X-ray beam spectrum. The effects of wedge filters presented are considered in Figure 4b. Namely, the uniformity of the effective quality distribution Q_eff_ implies much better uniformity from the center to the right side of the target when a similar effect is observed from the center of the target to the left side after applying the left wedge (Figure 4b).

As the scope of the experiment was to verify whether the LuAG:Ce crystal detectors give different responses with varying radiation spectrums, we exposed them to both open beams and several wedged beams. The position of the detector relative to the beam central axis (CAX) determines the degree of spectrum modification. Since the samples have been exposed separately, we were able to make the experiment independently of the dose by calculating and setting the required time. All the samples cumulated 2 Gy of dose, and the accuracy of such dose delivery was 0.3%.

## 4. Experimental Results

### 4.1. Cathodoluminescence and Thermoluminescence Spectra of LuAG:Ce Crystal Detectors

**Figure 5:**
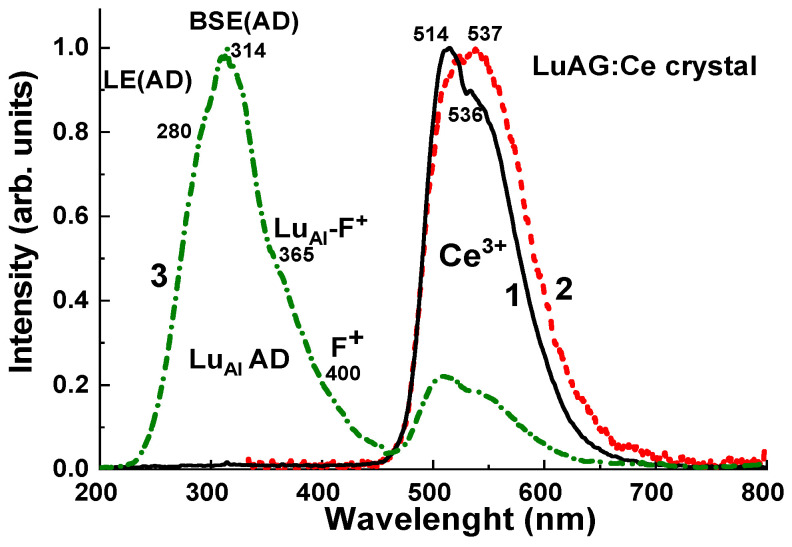
Normalized CL spectra (1) at RT and TSL spectra (2, 3) of LuAG:Ce crystals, irradiated by X-rays with a dose of 2 Gy, and recorded using BG 39 green filter (2) and UV 340 filter (3) during heating in the range of main TL peaks at 240–280 °C.

The CL spectrum of the selected sample of LuAG:Ce crystal at RT is shown in Figure 5, curve 1. The dominant emission band in the green range with two sub-bands peaking between 500–600 nm corresponds to the Ce^3+^ 5d^1^-4f (^2^F_5/2; 7/2_) radiative transitions in the LuAG host. In the UV range, the low intensive bands correspond to the emission enters caused by the Lu_Al_ ADs, namely to the luminescence of excitons localized (LE) and bound with Lu_Al_ AD (LE (AD) and BSE (AD) centers) [26,27,28,29].

**Figure 6:**
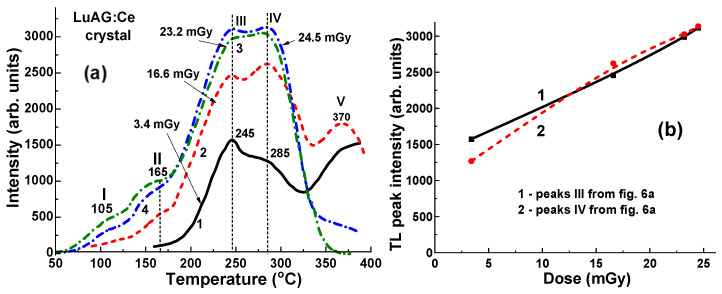
(**a**) Dependence of TL peak intensity and peak position for the LuAG crystal detector on the dose of irradiation in 3.4 to 24.5 mGy range by continuous X-rays, operating at 40–140 kV range. (**b**) Dependence of TL intensity on the dose of irradiation for the LuAG:Ce detector.

**Figure 7:**
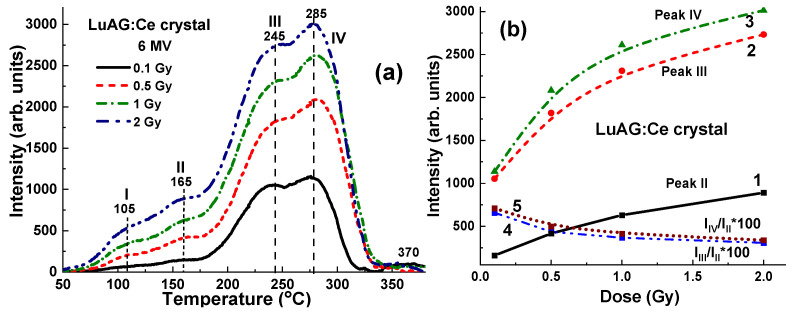
Dependence of TSL peak intensity and peak position for LuAG:Ce crystal detector on dose of irradiation by unfiltered 6 MV X-ray photons from Clinac 2300 accelerator; (**b**) dependence of TL intensity on irradiation dose for LuAG:Ce crystal detector for peaks II, III and IV from (**a**).

**Figure 8:**
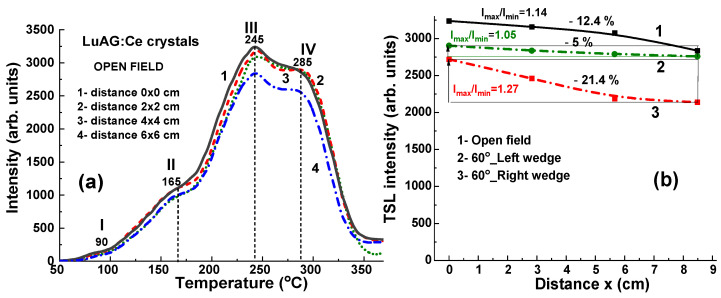
(**a**) TL glow curves for LuAG:Ce crystal detectors irradiated by 6 MV X-ray photons from a Clinac 2300 accelerator in open field mode; (**b**) dependence of intensity of the III (curve 1) and IV TL peaks (curves 2 and 3) on the distance x between sample and center of target (see Figure 1).

**Figure 9:**
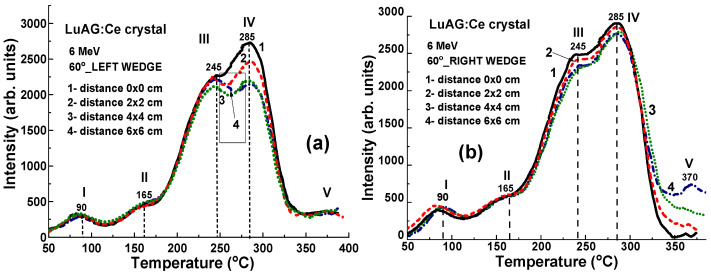
TL glow curves for LuAG:Ce crystal detectors irradiated by 6 MV X-ray photons from Clinac 2300 accelerator using additional 60°_Left (**a**) and 60°_Right (**b**) wedges (see Figure 4).

**Figure 10:**
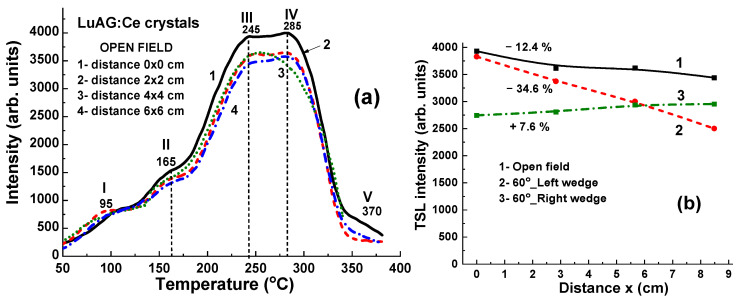
(**a**) The set of TL glow curves illustrates the response of LuAG:Ce crystal detectors to irradiation by 15 MV X-ray photons from a Clinac 2300 accelerator operating in open-field mode. (**b**) The intensity of the III TL peak is plotted against the distance (x) between the sample and the centers of the target, as shown in Figure 1 in the cases of open field mode (curve 1) and using 60°_Left (curve 2) and 60°_Right (curve 3) wedges.

**Figure 11:**
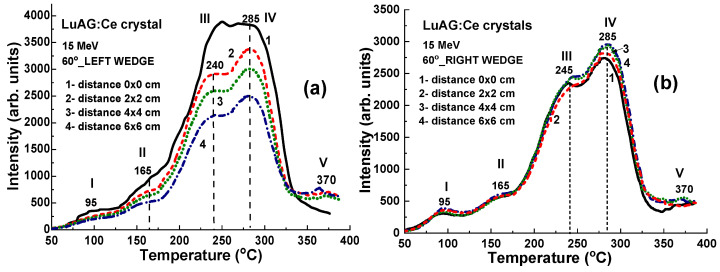
TL glow curves for LuAG:Ce crystal detectors irradiated by 15 MV X-ray photons from a Clinac 2300 accelerator using additional wedges: 60°_Left (**a**) and 60°_Right (**b**). Additionally, fragment Figure 10b depicts the dependence of the intensity of the III TL peaks on the distance x between the sample and the center of the target (refer to Figure 1) in the case of 60°_Left (curve 2) and 60°_Right (curve 3) wedges.

The TSL spectra of LuAG:Ce crystal recorded using green BG 29 and UV 340 filters during heating of the sample in the 200–350 °C range corresponding to the main III and IV TL peaks of this crystal (Figure 6, Figure 7, Figure 8, Figure 9, Figure 10 and Figure 11) are presented in Figure 5, curves 2 and 3, respectively. The sample was preliminary irradiated by an X-ray source with a dose of 2 Gy. The TSL spectrum, recorded using a green BG 39 filter, presents the dominant Ce^3+^ luminescence band caused by the 5d^1^-4f (^2^F_5/2; 7/2_) transitions of Ce^3+^ ions. It is interesting that the maximum of the TSL emission band is notably red-shifted in comparison with the Ce^3+^ band in the CL spectrum due to higher temperature of registration of CL (RT) and TSL (200–350 °C) spectra, respectively. Meanwhile, the TSL spectrum recorded using a UV 340 filter (curve 3), apart from the low-intensive Ce^3+^ emission band in the green range, also shows the dominant complex emission band in the UV range caused by the superposition of the emission bands of LE (AD) and BSE (AD) centers peaked approximately at 280 and 314 nm, respectively, and bumps at 364 and 400 nm, most probably corresponding to the luminescence of the defect LuAl-F^+^ and F^+^ centers [26,27,28,29,30,33]. 

The above-mentioned results confirm that the LuAG:Ce crystals possess a large concentration of the intrinsic defects (Lu_Al_ ADs and oxygen vacancies), which act as emission and trapping centers and, for this reason, can be used as *effective TL detectors* (see Section 4.2, Section 4.3, Section 4.4, Section 4.5). The recombination of the electrons from Lu_Al_ AD and F^+^-related centers with holes localized at Ce^3+^ ions results in intensive TL in the green range (Figure 6, Figure 7, Figure 8, Figure 9, Figure 10 and Figure 11). 

### 4.2. Dependence of TL Intensity of LuAG:Ce Crystal Detector on the X-ray Dose

Figure 6 illustrates the TL glow curves obtained from a chosen sample of LuAG:Ce crystal detector. The detector was previously exposed in an open field configuration using a conventional X-ray source to doses ranging from 3.4 to 24.5 mGy (0.0034–0.0245 Gy). This X-ray source operated with variable acceleration potential between 40 and 140 kV and current ranging from 200 to 400 mA. Despite the relatively low radiation dose and small sample size, the TL intensity of the LuAG:Ce detector remains notably high. However, all TL curves exhibit an asymmetric shape, with peak positions closely clustered within the 50–400 °C range (Figure 6a). The asymmetric shape of the glow curves is caused by the overlapping of five main peaks around 105 °C (I), 165 °C (II), 245 °C (III), 285 °C (IV), and 370 °C (V). The asymmetrical structure of glow curves is mainly caused by the high overall intensity of overlapping main III and IV TL peaks, even at such a low dose of radiation, and their different responses to the values of irradiation dose. There is also some influence of high-temperature blackbody radiation on the intensity V peak at 370 °C, especially at low irradiation doses of the sample (Figure 6, curves 1–4).

The dependence of TL intensity on the dose of irradiation in the 3.4–24.5 mGy range for the LuAG:Ce crystal detector for the main dominant peaks III (1) and IV (2) are shown in Figure 6b. These relationships show two different slopes in the 3.4–24.5 mGy range, which indicate the different nature of the trapping centers responsible for these TL peaks. However, of the most importance here is the practically linear dependence of the main TL peak’s intensity on the irradiation dose in the mentioned dose range. This means that the LuAG:Ce crystals can be used as a suitable dosimeter for estimation of the beam uniformity at typical radiation doses in radiology applications starting even from 1 mGy.

We have also investigated the dependence of the TL response of the LuAG:Ce crystal detector on the dose of unfiltered X-ray irradiation from a 6 MV linac in the range of therapeutic dose from 0.5 to 2 Gy (Figure 7a). All TL curves show a symmetrical shape and close peak positions in the range of 50–400 °C at 105 °C (I), 165 °C (II), 245 °C (III), 285 °C (IV), and 370 °C (V) (Figure 7a). The dependence of the TL intensity on the irradiation dose in this range for the main dominant peaks at 165 °C (II), 245 °C (III), and 285 °C (IV) is shown in Figure 7b. However, it is worth noting here that the intensity of the main TL II, III, and IV peaks shows quasi-nonlinear dependences on the radiation dose with different slopes in the 0.5–2 Gy range (Figure 7b, curves 1–3, respectively). Moreover, the ratio between the intensity of 165 °C (II)/245 °C (IV) peaks and 165 °C (II)/285 °C (IV) peaks also does not linearly decrease with an increase in the dose of irradiation, with this ratio being equal to about 3.1–3.4 at a dose of 2 Gy (Figure 7b, curves 4, 5). 

### 4.3. Results for 6 MV X-ray Irradiation

Figure 8 illustrates the TL glow curves for a set of LuAG:Ce crystals, which were irradiated by 6 MV X-ray photons from a Clinac 2300 accelerator. The irradiation was performed with a dose of 2 Gy in the open field mode without the use of wedges. As depicted in Figure 8a, the TL glow curves exhibit a comparable shape and closely clustered peak positions at 90 °C (I), 165 °C (II), 245 °C (III), and 285 °C (IV). It is also interesting to note here that the main TL peak in the TL glow curve after X-ray irradiation in the open field mode is the third peak (III) at 245 °C (Figure 8a) as compared to the case of irradiation with 60°_Left and Rith 60°_wedges, where the dominated fourth (IV) TL peak is observed at 285 °C (Figure 9). However, the main variations in TL peak intensities are due to differences in the energy of the X-ray radiation at various points on the target. Specifically, a higher intensity of the third (III) TL peak is observed in the sample at the center of the target, while the lowest intensity of this peak, which is 12.4% smaller, is found in the sample located approximately 8.5 cm from the center of the target (Figure 8a, curve 1). 

The use of additional wedges of various shapes can significantly affect the uniformity of the radiation dose and strengthen or weaken the effect of the flattening filter (Figure 9). In particular, the use of the 60°_Left left wedge particularly enhances the changes in the intensity of the TL glow peaks (Figure 8b and Figure 9a, curve 2). Conversely, the use of a 60°_Right wedge significantly reduces the TL intensity deviation between samples placed at different locations on the target (Figure 8b and Figure 9b, curve 3).

The corresponding relationships between the third (III) TL intensity of LuAG crystals and the distance x from the sample positions to the center of the target are depicted in Figure 8b, represented by curves 2 and 3, for the cases of using 60°_Left and 60°_Right wedges, respectively. In the case of 60°_Left wedge, the ratio I_max_/I_min_ of TL glow peak intensity of samples in the center and border positions is 1.27 times higher in comparison with the significantly smaller difference in the intensity (by 1.05 time) of these peaks in the case using a 60°_Right wedge.

### 4.4. Results for 15 MV X-ray Irradiation

Figure 10a depicts TL glow curves for a set of LuAG:Ce crystal detectors irradiated by 15 MV X-ray photons from a Clinac 2300 accelerator at a dose of 2 Gy in the open field mode, without wedges. When exposed to 15 MV X-ray photons, the TL glow curves exhibit similar dependencies on the localization of LuAG:Ce crystals in the target, as shown in both Figure 10a,b. This is in contrast to the behavior observed under irradiation by 6 MV X-rays in the open field mode, as illustrated by Figure 8a,b. One necessarily noted also here that the main TL peak in the TL glow curve after 15 MV X-ray irradiation in the open field mode is the IV peaks at 285 °C (Figure 10a) as opposed to 6 MV X-ray irradiation where the dominant III TL peak at 245 °C is observed (Figure 8a). Furthermore, in the case of 15 MV irradiation, the intensity of TL peaks is quite large (by 1.25 times) (Figure 10a) in comparison with 6 MV irradiation (Figure 8a) at the same doses.

Significant variations in the highest TL peak intensities are observed following 15 MV photon irradiation, attributed to differences in the energy of the X-ray radiation at various points on the target. A higher intensity of the III TL peak is manifested in the sample in the center of the target when the smallest (at 12.4%) intensity of this peak is found in the sample located at the largest (~8.5 cm) distance from the target center (Figure 10a, curve 1). 

When the high-energy part of 15 MV X-ray radiation is absorbed by the 60°_Left and 60°_Right wedges (depicted in Figure 11a and Figure 11b, respectively), the LuAG:Ce detectors exhibit high TL intensity. Such crystal detectors prove suitable for accurately measuring the uniformity of the X-ray beam. Specifically, when using the 60°_Left wedge, the TL intensity of LuAG:Ce detectors decreases significantly (by 34.6%) as the distance (x) between the sample and the target centers increases (as shown in Figure 10b, curve 2). Conversely, when using the 60°_Right wedge, the TL intensity of detectors only experiences a slight increase of 7.6% with the distance of sample localization (as depicted in Figure 10b, curve 3).

### 4.5. Results for ^60^Co γ-ray Irradiation

**Figure 12:**
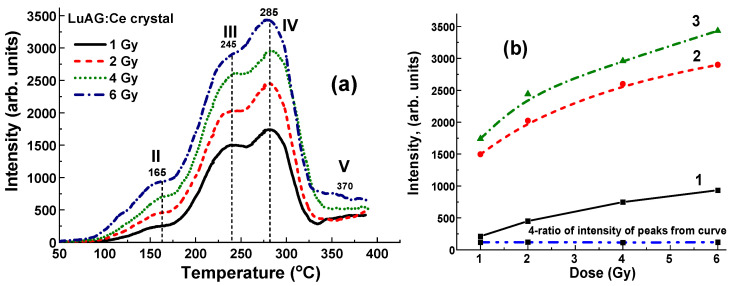
(**a**) Dependence of intensity of TL glow curves for LuAG:Ce crystal detector on the dose of irradiation in the 1–6 Gy range by γ-rays from ^60^Co source; (**b**) intensity of TSL glow peaks of LuAG:Ce crystal at 165 °C (II)—curve 1, 245 °C (III)—curve 2, and 285 °C (IV)—curve 3 and I_245_/I_285_ ratio of intensity of these peaks (curve 4) on dose of γ-rays irradiation by ^60^Co source.

Figure 12 illustrates the TL glow curves for LuAG:Ce crystal detectors irradiated by γ-rays with energies of 1.17 and 1.33 MeV from ^60^Co sources, with doses ranging from 1 to 6 Gy in the open field mode. It is noteworthy that in the case of γ-rays irradiation from a ^60^Co source, the flattening filter is absent, unlike X-ray irradiation (as seen in Figure 8 and Figure 10). All the TL curves after ^60^Co irradiation (Figure 12) show the well distinguished and symmetrical structure consisting of four main peaks at 165 °C (II), 240 °C (III), 285 °C (IV), and 380 °C (V). The low-temperature peaks in the 90–105 °C range are not observed, most probably due to the feeding effects caused by the long period after γ-irradiation and TL readout (three days). Interestingly, the intensity of all TL glow peaks shows quite nonlinear dependence on the dose of radiation with different slopes in the 1–6 Gy range (Figure 12b, curves 1–3, respectively). Most probably, the thickness of the sample (1 mm) is not optimal for recording γ-rays with energy of 1.17 and 1.33 MeV, and it should be increased to at least 2–3 mm. At the same time, the ratio between the intensity of the main 245 °C (III)/285 °C (IV) peaks equal to 1.14–1.18 is constant with respect to the dose of irradiation (Figure 12, curve 4). 

Furthermore, the intensity of TL of LuAG:Ce crystal samples in the case of ^60^Co irradiation (Figure 12a) is comparable to the TL intensity of these detectors after X-ray radiation with 6 MV and 15 MV accelerators (Figure 8a and Figure 10a), confirming the universality of such type of detectors for considering medical applications. Most likely, the TSL response under γ-rays excitation depends not only on the radiation dose but also on the thickness of the LuAG:Ce crystal detectors. 

## 5. Discussion

As was mentioned in the Introduction, due to the very high energies of X-ray beams produced by the linac with 6 MV and 15 MV acceleration potentials, application of the conventional low-density and low-Z_eff_ TL detectors based on LiF or Al_2_O_3_:C is limited to the investigation of uniformity of X-ray beam and other high-energy radiations. For this reason, efficient TL detectors based on YAG:Ce have recently been proposed to study the uniformity of the therapeutic X-ray beam from a 6 MV and 15 MV linac source and the detection of γ-rays from a ^60^Co source [15]. However, compared to YAG:Ce TL detectors, LuAG:Ce crystals demonstrate superior performance in terms of TL efficiency for determining X-ray beam uniformity both for low-energy 6 MV and high-energy 15 MeV Linacs. Namely, the TL intensity of YAG:Ce crystal detectors is substantially less for the registration of X-ray radiation generated by an accelerator with a nominal energy of 15 MV in open field mode, compared to 6 MV radiation as well as for the registration of γ-rays produced by a ^60^Co source with energies of 1.17 and 1.33 MeV. This is due to the relatively low density (ρ = 4.5 g/cm^3^) and effective atomic number (Z_eff_ = 35) of the YAG material [34]. Additionally, the intensity of the main TL peak of YAG crystals in the 290–300 °C range changes slightly non-linearly with a dose of higher energy radiation from a ^60^Co source and X-rays from a 15 MV accelerator. For this reason, in order to investigate the uniformity of the X beam in a higher energy (15 MV) linac in radiotherapy, YAG:Ce crystal detectors should be replaced or combined with a heavier analog, i.e., LuAG:Ce garnet crystals, which have a density of ρ = 6.73 g/cm^3^ and effective atomic number Z_eff_ = 62. This makes LuAG:Ce crystals TL detectors potentially more suitable for applications with higher energy X and γ-rays.

The above-mentioned results of the application of the crystals of “heavy” LuAG:Ce garnet with ρ = 6.73 g/cm^3^ and Z_eff_ = 62 as TL detector are very encouraging. The obtained results on X-ray irradiation from typical 6 MV and 15 MV Linac accelerators (Figure 8, Figure 9, Figure 10 and Figure 11) and γ-ray irradiation by ^60^Co source (Figure 12) confirm that LuAG:Ce crystals are fully consistent TL material for the investigation of beam uniformity at therapeutic radiation treatment by γ-rays with energies above 1 MV as well as by high-energy 6 MV and 15 MV X-ray phonons using flattening filter both in the open field mode or in the modes with the 60°_Left and 60°_Right wedge filters.

The collective findings depicted in Figure 6, Figure 7, Figure 8, Figure 9, Figure 10 and Figure 11 indicate that both the shape and intensity of the TL glow curves are significantly influenced by not only the dose but also the energy of X-ray radiation. Specifically, the LuAG: Ce crystal detectors exhibit high TL intensity under both 6 MV and 15 MV X-ray irradiation in the open field mode, with minimal deviation (not exceeding 12.5%) in the intensity of the main peaks within the 245–285 °C range, even as the position of the samples relative to the center of the target changes due to the hardening of the central part of the X-ray beams. Moreover, such a deviation can be increased to −(21.4–34.5%) or strongly decreased to +(5–7.7%) using 60°_Left and 60°_Right wedge filters, respectively. 

Following the main results of our previous work [14] on the application of “lighter” YAG:Ce crystal detectors with smaller density ρ = 4.5 g/cm^3^ and Z_eff_ = 39, we consider the universal TL composite detectors based on the LPE-grown YAG:Ce film/LuAG:Ce crystal epitaxial structures for estimating of the beam uniformity obtained by accelerators working with both 6 MV and 15 MV NAP and γ-rays of ^60^Co and ^192^Ir sources. Furthermore, the LPE growth of such a type of epitaxial structure is also an important step in the development of composite scintillators based on garnet compounds for measuring the radiation dose of low-penetration α-particles and ^7^Li ions and high-penetrating γ-rays in the boron-capture-neutron therapy (BNCT) at the tumor treatment procedure [35,36].

## 6. Conclusions

In this work, the thermoluminescence (TL) properties of LuAG:Ce garnet crystal detectors were used for radiotherapy applications. The primary goal of the research was to establish the dose and energy spectra of therapeutic radiation sources. This included clinical radiotherapy X-ray beams operating at nominal accelerator potentials of 6 MV and 15 MV, as well as γ-rays emitted by the ^60^Co source, characterized by energies of 1.17 and 1.33 MeV.

The LuAG:Ce crystal detector demonstrates an exceptionally high TL light yield and exhibits a nearly linear dependence of the intensity of the main TL peaks at 245 °C and 285 °C on the dose of X-ray irradiation within the 3–25 mGy range. These X-rays are generated by a continuous X-ray source with various acceleration potentials ranging from 40 to 160 kV. Moreover, the intensity of the main TL peaks of LuAG:Ce crystals within the 245–285 °C range also shows good proportionality with the dose of higher-energy X-ray radiation produced by a 6 MV accelerator in the 0–2 Gy range, as well as with γ-ray radiation from a ^60^Co source in the 1–6 Gy range.

Additionally, we determined that TL detectors, manufactured from an identical LuAG:Ce crystal, are effective dosimeters for assessing the uniformity of the X-ray beam in a 6 × 6 cm^2^ target field during therapeutic radiotherapy, using irradiation with a dose of 2 Gy from a clinical accelerator operating both with 6 MV and 15 MV potentials. Namely, using the LuAG:Ce crystal detectors, a small deviation of around 12.4% in the intensity of the main peaks at the 245–285 °C range on the position of the samples with respect to the center of the target has been found after 6 MV and 15 MV X-ray irradiation with flattening filter in the open field mode due to hardening of the central part of X-rays beams. However, such deviation can be increased to −(21.4–34.5) % or strongly decreased to +(5–7.7)% using 60°_Left and 60°_Right wedge filters, respectively.

Future works on the development of universal TL composite detectors based on the epitaxially grown YAG:Ce/LuAG:Ce structures are considered nowadays as actual tasks of radiotherapy applications. 

## Data Availability

The original contributions presented in the study are included in the article, further inquiries can be directed to the corresponding authors.
